# Focused sonographic examination of the heart, lungs and deep veins in acute admitted patients with respiratory symptoms

**DOI:** 10.1186/1757-7241-20-S2-P22

**Published:** 2012-04-16

**Authors:** Christian B Laursen, Erik Sloth, Jess Lambrechtsen, Annmarie Touborg Lassen, Finn Rasmussen

**Affiliations:** 1 Research Unit at The Department of Respiratory Medicine, Odense University Hospital, Denmark; 2 Department of Anaesthesia and Intensive Care, Aarhus University Hospital, Skejby, Denmark; 3 Department of Medicine, Odense University Hospital, Svendborg, Denmark; 4 Acute Medical Admission Unit, Odense University Hospital, Denmark

## Background

Acutely admitted patients with respiratory symptoms remains a diagnostic challenge. At the primary evaluation the clinician has to rely on the clinical examination when initiating treatment and further diagnostic work up. Several studies have questioned the diagnostic performance of the clinical examination. In addition, most of the diseases, which are commonly seen in patients with acute respiratory symptoms, can be diagnosed using sonography. Sonography could be integrated as a part of the primary evaluation, potentially improving the diagnostic performance. We therefore evaluated the use of sonographic examination of the heart, lungs and deep veins, performed within one hour of the primary evaluation, in acute admitted patients with respiratory symptoms.

## Methods

We performed a prospective cross sectional blinded observational study, conducted in a medical emergency department. Patients were included if one or more of the following symptoms or clinical findings were present: respiratory rate > 20, saturation < 95 %, oxygen therapy initiated, dyspnoea, cough or chest pain. Within one hour after the primary evaluation, focused sonography of the heart, lungs and deep veins was performed by a physician blinded to patient history and the results of the evaluation. If the sonography identified any predefined acute life threatening conditions, which had been missed at the primary evaluation, the physician in charge was informed. Other findings of the sonographic examination remained blinded.

## Results

We identified and screened 342 patients of whom 139 patients were included in the study. The sonography feasibility was 97 %. In 21 (15 %) patients sonography identified an acute life threatening condition missed at the primary evaluation. Of these, 10 had left-sided heart failure with pulmonary oedema, 1 had pericardial effusion, 1 had massive pleural effusion, 5 had empyema and 4 had pulmonary embolism.

## Conclusion

Focused sonography of the heart, lungs and deep veins is a highly feasible and non-invasive bedside method. In acute admitted patients with respiratory symptoms, it may help the clinician diagnose acute life threatening conditions, which would otherwise have been missed at the primary evaluation. Further studies are needed to assess the diagnostic accuracy and evaluate the consequences of including all sonographic findings as a part of the primary evaluation.

